# Biological control of the Asian chestnut gall wasp in Portugal: Insights from a mathematical model

**DOI:** 10.1371/journal.pone.0254193

**Published:** 2021-07-21

**Authors:** Carlos Balsa, Albino Bento, Francesco Paparella

**Affiliations:** 1 Research Centre in Digitalization and Intelligent Robotics (CeDRI), Instituto Politécnico de Bragança, Bragança, Portugal; 2 Mountain Research Center (CIMO), Instituto Politécnico de Bragança, Bragança, Portugal; 3 Division of Science and NYUAD Research Institute, New York University Abu Dhabi, Abu Dhabi, UAE; University of Carthage, TUNISIA

## Abstract

In recent years, the Asian gall wasp *Dryocosmus kuriphilus* has invaded chestnut trees and significantly affected the Portuguese chestnut production. Studies in other countries, such as Japan or Italy, have shown that the parasitoid *Torymus sinensis* can successfully achieve biological control of *D. kuriphilus*. Mathematical models help us to understand the dynamics of the interaction between the pest *D. kuriphilus* and its parasitoid *T. sinensis* and, consequently, they can help to implement measures that enhance crop pest management. In this work, the evolution of the density of *D. kuriphilus* and *T. sinensis* across time and space is studied through the numerical solution of models that include parameters based on observations made in Portugal. Simultaneous releases of the parasitoid are simulated at various locations and at different times. The results indicate that, in the case of a small and homogeneous orchard, biological control can be effective, but, in the case of extensive domains, the pest control is much more difficult to achieve. In order for biological control to be efficient, it is necessary to implement, in each chestnut-producing region, a collective strategy based on the annual monitoring of infestation levels.

## Introduction

**Fig 1:**
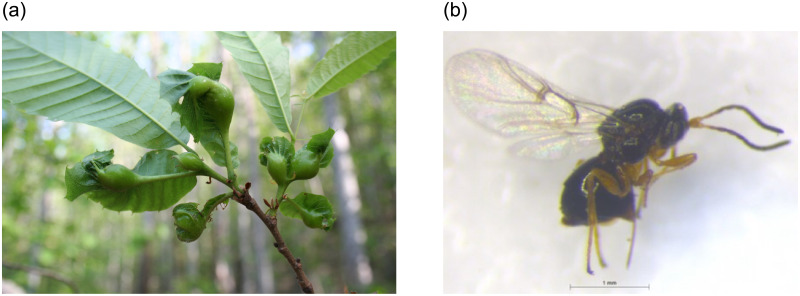
Galls induced by *Dryocosmus kuriphilus* (a) and *Dryocosmu kuriphilus* adult (b).

The Asian chestnut gall wasp, *Dryocosmus kuriphilus* Yasumatsu (Hymenoptera: Cynipidae), is a pest that disrupts the growth of chestnut trees and limits fruit production [1, 2]. Indeed, this insect lays its eggs in the buds of chestnuts in early summer and the larva spends all winter in this bud. In the following spring this induces the formation of galls on the buds and on the leaves of the tree (Fig 1). Therefore, attacks by this pest can significantly reduce growth, fruiting and consequently the production of chestnut trees, with further negative economical effects [3]. The reduction in chestnut production can reach 85% in cases of high infestation levels [4].

*Dryocosmus kuriphilus*, native to China, spread to other Asian countries and North America in the last century. At the beginning of this century, it arrived in Europe, where it was observed for the first time in Italy in 2002. In 2005, the pest was detected in France from where it spread to other countries, including Portugal (2014). Recent research suggests that the spread of *D. kuriphilus* populations in Europe could be the result of a single introduction of a Chinese founder population that subsequently spread rapidly across Europe [5].

**Fig 2:**
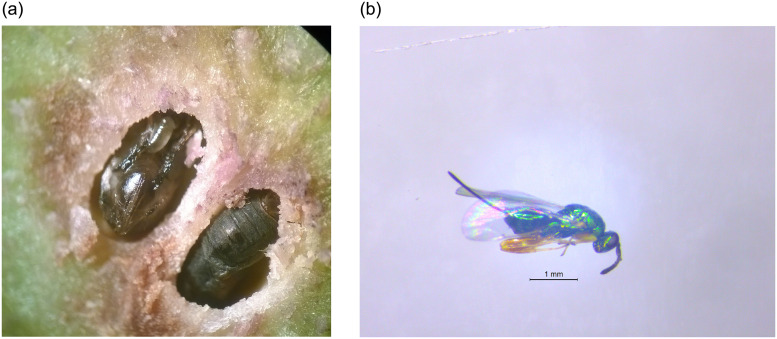
*Dryocosmus kuriphilus* pupae with parasitoid larvae (a) and adult parasitoid *Torymus sinensis* (b).

Solving this phytosanitary problem by adopting the most effective pest management options is very important for the economy of this sector, specially for countries that are big producers, like Portugal, with an estimate of 30,000 to 35,000 tons of annual chestnut production. Biological control is considered the most effective method of control of *D. kyriphilus* [6]. It was firstly implemented in Japan, where the researchers performed tests with several species of parasitoids and discovered that *Torymus sinensis* Kamijo (Hymenoptera: Torymidae), a parasitoid that also came from China, could be used to effectively control the pest. This parasitoid is reported to be highly specific and its life cycle is synchronized with that of the Asian gall wasp. *Torymus sinensis* lays its eggs in the larvae (or pupae) of *D. kyriphilus* (see Fig 2); this prevents them from developing and thus to later attack the chestnut tree and to further reproduce [7]. Besides Japan (see for instance [8–10]), biological control programs, based on the importation of *T. sinensis* from its native area, have been carried out in different American and European countries.

A mathematical model of the biological control of the chestnut Asian gall wasp by *T. sinensis* was developed by F. Paparella et al. [11]. This is a spatially-extended host-parasitoid model that explicitly describes the seasonal dynamics of both the host and of the parasitoid populations. In its full form, it is embodied by a set of coupled partial differential equations of the reaction-diffusion type, whose unknowns are the densities of adult and eggs of both species. The multi-year dynamics is reconstructed by using the end-of-season densities of year *n* as initial conditions for the year *n*+1. When spatial homogeneity can be assumed (e.g. in the case of small orchards), the model simplifies to a set of ordinary differential equations, whose dynamics bear some resemblance to predator-prey models [12], although the interaction between the adults of the two species remains always indirect, because predation occurs at the larval stage, inside chestnut galls. The model can be adapted to a specific location by tuning the value of the parameters that characterize the interactions of both the host and the parasitoid with the local environment (e.g. the overwintering mortalities).

Paparella et al. [11] had to deal with the uncertainty associated with the values of certain parameters, namely, the overwintering survival rates of the two species and the ratio of the diffusive coefficients of the two species. The model solutions were found to be strongly affected by the values of these parameters. The overwintering survival rate is defined as the fraction of laid eggs that successfully originate adult females in the next year. That of *D. kuriphilus* can be close to 100% thanks to parthenogenetic reproduction, while that of the sexually reproducing *T. sinensis* cannot exceed 50% because male and female individuals emerge with roughly the same probability from fertilized eggs [13]. Many factors may reduce these theoretical maximal rates, most importantly the level of natural parasitism. Despite the uncertainty, it is common to assume that in Europe, *D. kuriphilus* and *T. sinensis* overwintering survival rates are near 90% and 45%, respectively [11].

The mortality of *D. kuriphilus* larvae is mainly due to the presence of parasitoids (see, for instance, [14, 15]). Using a simplified mathematical formulation, valid in the case of spatial homogeneity, Paparella et al. [11] showed that low overwintering survival rates result in a cyclical evolution of population densities for the two species, very similar to a typical predator-prey cycle, with cycles spanning only a few years, and small-amplitude fluctuations in the density of both populations. Under these conditions, biological control is not achieved, because *T. sinensis* is unable to reduce the population density of *D. kuriphilus* to levels sufficiently low as to be considered extinct, or, at least, to persistently keep it at non damaging levels. On the contrary, with high overwintering survival rates the model shows longer cycles and extreme drops in the population density of both species, which, in reality, would correspond to local extinction, that is, to the achievement of biological control.

In northwestern Portugal, a high number of native parasitoids were reported parasitizing *D. kyriphilus* [16, 17]. The role of native parasitoids, that normally attack cynipid gall wasps on oaks, is currently under investigation within the scope of biological control programs. In this work, we assess the effect of natural parasitism (as quantified by the overwintering mortality parameter) on the possibility of achieving biological control of *D. kuriphilus* through *T. sinensis*, in the specific setting of Portugal. We perform several numerical simulations with values of the overwintering survival rates lower than that normally considered appropriate for the rest of Europe (90%).

The diffusive ratio, defined as the quotient between the diffusion coefficient of the parasitoid over that of the gall wasp, reflects the relative dispersion capacity of the two species. This coefficient is taken to be less than one because *D. kuriphilus* appears to have a greater dispersion capacity than *T. sinensis*. As a result, the gall wasp is able to move to areas where the parasitoid does not occur in sufficient number to prevent its establishment [11]. This makes biological control action difficult, and it is necessary to define appropriate strategies to increase its effectiveness.

A previous preliminary study suggested that the reinjection of the parasitoid can contribute to the reduction of the number of years during which *D. kuriphilus* is present at its maximum population density [18]. However, a detailed search for optimal pest management strategies has not been done yet. In addition, the definition of an optimal strategy implies adapting the model to regional conditions. In Portugal, it was reported that *D. kuriphilus* can disperse through active fly, with an average rate of dispersion estimated to be 8 km by season [16]. Regarding *T. sinensis*, there is no specific studies in Europe, but it is common to assume that its dispersion capacity is much lower than that of *D. kuriphilus* and does not exceed 2 km per year, as reported by Toda et al. for Japan [19].

These dispersion parameters, along with different gall wasp overwintering survival rates, are used in the model to simulate biological control scenarios applicable to the chestnut–producing regions in Portugal, whose chestnut–cultivated areas are characterized by having surfaces less than 25 km^2^. Simulations of simultaneous releases of the parasitoid are made in various places and at different times. The effects of the dimension of the forest area, the periodic repetition of the releases and the distance between them are studied. The results obtained allow us to draw important conclusions about the factors that enhance biological control and help to define effective strategies for achieving it in Portugal.

The paper is organized as follows. In Section **Biological cycle of the pest and its parasitoid** there is a short description of the biological cycles of the two insects involved in the modeling. Section **Modelling the Temporal Evolution of** ***D. kuriphilus*** **and** ***T. sinensis*** **Population Densities** introduces the spatially-homogeneous mathematical model and presents the results of numerical simulations that enable the estimation of the density of the two species over the time, considering different overwintering survival rates of the Asian gall wasp. Section **Space-Dependent Model** is devoted to the study of the spatial variation of the two species over the years and to the analysis of some strategies that can improve the efficiency of biological control. Section **Final Considerations** wraps up the paper with some final considerations.

## Biological cycle of the pest and its parasitoid

**Fig 3:**
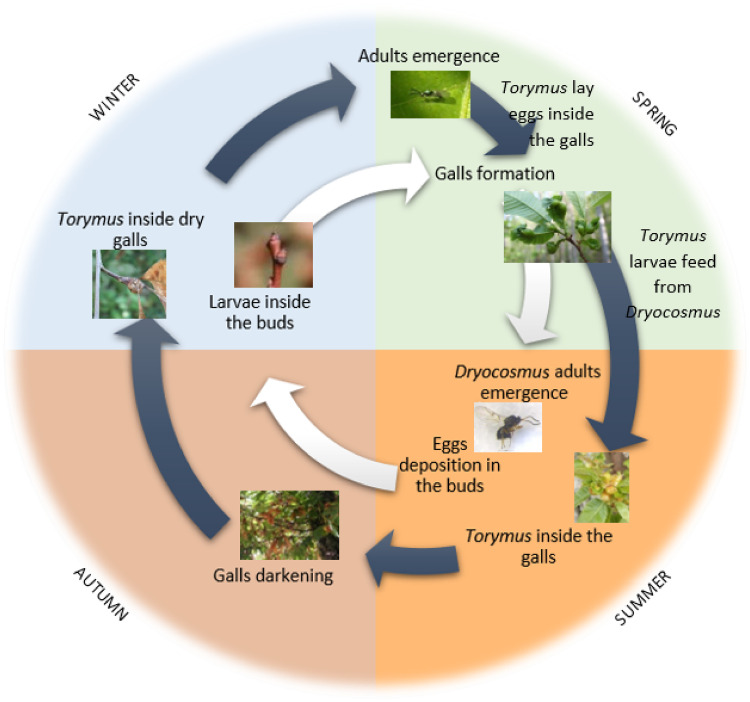
Biological cycle of *Dryocosmus kuriphilus* and *Torymus sinensis*.

The life cycle of *D. kuriphilus* starts in early summer (June—July), with the emergence of the adults, which, given their thelytokous nature, do not require mating, and are able to immediately start oviposition, that occurs exclusively inside the chestnut tree buds. The adults have a short life span of less than 10 days, during which they can lay more than 100 eggs in groups of 3 to 5 per bud [10, 20]. The eggs take about 30 to 40 days to hatch, but the larva will stay dormant and imperceptible during winter, in the buds of chestnut trees [21]. At the beginning of the following spring, when the chestnut tree starts its vegetative activity, the larvae start to move, inducing gall formation, of which they will feed, for approximately 30 days. After that, pupation starts, which will last until the beginning of summer, when the adult insects emerge. Fig 3 shows schematically the life cycle of both *D. kuriphilus* and *T. sinensis*.

Depending on locality (altitude, exposure) and chestnut cultivar, pupation occurs from mid-May until mid-July [20]. *Dryocosmus kuriphilus* adults emerge from galls from the end of May until the end of July. This period corresponds to the eggs deposition season, the duration of which may extend up to 50 days [20].

*Torymus sinensis* is univoltine, with one generation per year, and haplodiploid, giving birth to haploid males from unfertilized eggs and diploid females from fertilized ones [22]. Females lay their eggs in early spring (March-April), inside the larval chambers of recently formed *D. kuriphilus* galls, usually one egg per host larva. If multiple eggs are laid in the same chamber, only one of them will grow to adulthood due to cannibalism among the hatched larvae [21, 23]. The larvae hatch quickly from the eggs and immediately start to feed from their host. At the end of the spring, they have already consumed the host and entered a dormant state until late winter, when pupation starts. They finally emerge in early spring, synchronous with the sprouting of chestnut trees and the formation of galls caused by *D. kuriphilus* [21, 23] (see Fig 3).

The life span of a *T. sinensis* *adult* can reach 40 or more days [8]. This depends mainly on the ambient temperature and the type and quantity of food available [8, 24]. The length of the egg deposition season for *T. sinensis* is normally set according to its lifespan [25]. Studies carried out in Portugal did not show the existence of natural enemies of this insect [16, 21].

## Modelling the temporal evolution of *D. kuriphilus* and *T. sinensis* population densities

In this Section, mathematical models that take into account the separate evolution of *D. kuriphilus* and of *T. sinensis*, over a single season, are introduced, followed by a complete model for the joint population dynamics of the two species over multiple seasons. Initially, we shall not take into account the spatial variability of population densities *i.e.*, it will be assumed that the two species are homogeneously distributed within a given territory, with no population fluxes through its boundaries. This homogeneous model, not explicitly dependent on space, enables to estimate the evolution over time of the density of the two species in a small and insulated orchard, and consequently evaluate the possibility of achieving biological control in relatively small–sized orchards.

### Model for *D. kuriphilus*

Let *U*_*n*_ be the population of adult gall wasps carrying eggs during the summer of the year *n*. Let *V*_*n*_ be the density of eggs laid in the chestnut buds.

Considering the density *β*_*max*_ of chestnut buds and *M* the maximum number of eggs to be laid per bud, then the maximum density of eggs laid is:

$$\begin{array}{c} V_{max}=M\beta_{max} \end{array}$$ (1)

Let *η* be the survival rate during the overwintering (proportion of eggs that survive the winter and originate larvae that successfully emerge as adults); this will depend on the geolocation of the species (*e.g.*, it will not be the same in Japan and Portugal). Let *T*_*d*_ be the length (days) of the egg deposition season. Assuming, for simplicity, a rate independent of time, emergence rate is:

$$\begin{array}{c} \text{emergence}\;\;\text{rate}=\frac{\eta V_{n-1}\left(T_{d}\right)}{T_{d}} \end{array}$$ (2)

Let *a* be the adult lifespan (*a* < *T*_*d*_), in days, and *t* one day of season *n*. Assuming, again, independence of time, the death rate is:

$$\begin{array}{c} \text{death}\;\;\text{rate}=-\frac{U_{n}\left(t\right)}{a} \end{array}$$ (3)

Finally, let *r*_*d*_ = *N*_*d*_/*a* be the number of eggs laid by a *D. kuriphilus* adult in the time unit under the most favorable conditions, with *N*_*d*_ being the maximum number of eggs that can be laid by an adult female. The laying rate is proportional to the product of the density of eggs that can be laid in a given location, which in the model is expressed as *V*_*max*_ − *V*_*n*_(*t*), by the density of the adult population:

$$\begin{array}{c} \text{egg}\;\;\text{deposition}\;\;\text{rate}=r_{d}\frac{V_{max}-V_{n}\left(t\right)}{V_{max}}U_{n}\left(t\right). \end{array}$$ (4)

The combination of all these quantities results in the following formulation that describes the evolution of *U*_*n*_ and *V*_*n*_ during the season of the year *n*:

$$\begin{array}{c} \left\{ \begin{array}{c} {\dot{U}}_{n}\left(t\right)=-\frac{1}{a}\frac{V_{max}-V_{n}\left(t\right)}{V_{max}}U_{n}\left(t\right)-\frac{1}{a}U_{n}\left(t\right)+\eta \frac{V_{n-1}\left(T_{d}\right)}{T_{d}}\\ {\dot{V}}_{n}\left(t\right)=\frac{N_{d}}{a}\frac{V_{max}-V_{n}\left(t\right)}{V_{max}}U_{n}\left(t\right) \end{array}\right. \end{array}$$ (5)

In Eq (5), the egg deposition rate, that is the only term of the right-hand side of the equation for *V*_*n*_, also appears in the equation for *U*_*n*_ with a minus sign and divided by *N*_*d*_. This term thus represents female individuals who have already laid all their eggs (*N*_*d*_) and, as such, are no longer part of the population that carries eggs (*U*_*n*_).

Non-dimensional field variables are used in the following. They are defined as: *u*_*n*_ = *U*_*n*_/(*ηV*_*max*_), *v*_*n*_ = *V*_*n*_/*V*_*max*_. Moreover, we define the non–dimensional time as $\tilde{t}=t/T_{d}$, and two non–dimensional parameters as *μ* = *T*_*d*_/*a* and *E*_*d*_ = *ηN*_*d*_. Consequently, the non-dimensional model for $\tilde{t}\in [0,\;1]$, together with the relevant initial conditions becomes:

$$\begin{array}{c} \left\{ \begin{array}{c} {\dot{u}}_{n}\left(\tilde{t}\right)=-\mu \left(2-v_{n}\left(\tilde{t}\right)\right)u_{n}\left(\tilde{t}\right)+v_{n-1}\left(1\right)\\ {\dot{v}}_{n}\left(\tilde{t}\right)=E_{d}\mu \left(1-v_{n}\left(\tilde{t}\right)\right)u_{n}\left(\tilde{t}\right)\\ u_{n}\left(0\right)=0\\ v_{n}\left(0\right)=0 \end{array}\right. \end{array}$$ (6)

Note that these ordinary differential equations, valid for the *n*−th year, are naturally coupled with the dynamics of the previous year through the (*n* − 1)−th year end–of–season egg density *v*_*n*−1_(1). The initial conditions simply state that at the beginning of the season there are no adults of *D. kuriphilus* nor any newly deposed eggs.

### Model for *T. sinensis*

To obtain the mathematical model for the evolution of *T. sinensis*, we define *P*_*n*_ as the population of the egg-carrying *T. sinensis* females in the year *n*, and *Q*_*n*_ as the density of eggs laid in the same year.

To estimate the egg deposition rate, as in the case of *D. kuriphilus*, let *r*_*t*_ be the number of eggs laid by each *T. sinensis* female in the unit of time under the most favorables conditions. In these conditions *r*_*t*_ ≈ *N*_*t*_/*T*_*t*_, with *N*_*t*_ being the maximum number of eggs that can be laid by an adult female during its lifespan *T*_*t*_. Thus, the egg laying rate is given by the product of egg-carrying *T. sinensis* female with the density of the sites where oviposition is possible:

$$\begin{array}{c} \text{egg}\;\;\text{deposition}\;\;\text{rate}=r_{t}\frac{\eta V_{n-1}\left(T_{d}\right)-Q_{n}\left(t\right)}{V_{max}}P_{n}\left(t\right). \end{array}$$ (7)

In Eq (7), the density of sites available for egg deposition is given by the difference between the density of gall wasp eggs laid during the previous season and turned into larvae (*ηV*_*n*−1_(*T*_*d*_)) and the density of *T. sinensis* eggs already laid.

Once combined, these quantities yield a set of equations describing the evolution of *P*_*n*_ and *Q*_*n*_ during the year *n*:

$$\begin{array}{c} \left\{ \begin{array}{c} {\dot{P}}_{n}\left(t\right)=-\frac{1}{T_{t}}\frac{\eta V_{n-1}\left(T_{d}\right)-Q_{n}\left(t\right)}{V_{max}}P_{n}\left(t\right)\\ {\dot{Q}}_{n}\left(t\right)=r_{t}\frac{\eta V_{n-1}\left(T_{d}\right)-Q_{n}\left(t\right)}{V_{max}}P_{n}\left(t\right) \end{array}\right. \end{array}$$ (8)

In Eq (8), the egg deposition rate appears also in the equation for *P*_*n*_ with a minus sign and divided by *N*_*t*_. This term thus represents individuals who have already laid all their eggs (*N*_*d*_) and, as such, are no longer part of the population of *T. sinensis* that carries eggs (*P*_*n*_). Contrary to the wasp model, the parasitoid model does not include any terms related either to the emergence or to the mortality of adult parasitoids. The reason is that *T. sinensis* adults are reported to emerge almost simultaneously and then live for an entire season, whose length coincides with the typical adult life span *T*_*t*_ [24], thus their reproductive ability is to a good approximation only limited by the availability of egg-deposition sites. In Portugal, the likelihood of death of an adult *T. sinensis* is so low (for lack of natural enemies) that it makes no sense to include an explicit mortality term.

While a mortality term for the adult phase appears to be unnecessary, *T. sinensis* is subject to death at the egg or larval stage. This is summarized by introducing a constant overwintering survival rate *γ* defined as the proportion of all eggs deployed in the previous year that originate adult females in the current year (*γ* < 0.5). This coefficient appears in the initial conditions for Eq (8), which are:

$$\begin{array}{c} \left\{ \begin{array}{c} P_{n}\left(0\right)=\gamma Q_{n-1}\left(T_{t}\right)\\ Q_{n}\left(0\right)=0 \end{array}\right. \end{array}$$ (9)

Defining the non-dimensional variables *p*_*n*_ = *P*_*n*_/(*ηγV*_*max*_), *q*_*n*_ = *Q*_*n*_/(*ηV*_*max*_), $\tilde{t}=t/T_{d}$, *τ* = *T*_*t*_/(*ηT*_*d*_) we obtain, for $\tilde{t}\in \left[0,\eta \tau \right]$ the non-dimensional model for *T. sinensis* of Eq (10).

$$\begin{array}{c} \left\{ \begin{array}{c} {\dot{p}}_{n}\left(\tilde{t}\right)=-\frac{1}{\tau }(v_{n-1}\left(1\right)-q_{n}\left(\tilde{t}\right))p_{n}\left(\tilde{t}\right)\\ {\dot{q}}_{n}\left(\tilde{t}\right)=\frac{E_{t}}{\tau }(v_{n}\left(1\right)-q_{n}\left(\tilde{t}\right))p_{n}\left(\tilde{t}\right)\\ p_{n}\left(0\right)=q_{n-1}\left(\eta \tau \right)\\ q_{n}\left(0\right)=0 \end{array}\right. \end{array}$$ (10)

where *E*_*t*_ = *γN*_*t*_ and *q*_*n*−1_(*ητ*) is the density of eggs laid by *T. sinensis* at the end of the previous season (*n* − 1). Note that in the new variables the initial condition is simply *p*_*n*_(0) = *q*_*n*−1_(*ητ*) because the coefficient *γ* is absorbed in the definition of *p*_*n*_.

### Complete spatially-homogeneous model

To have a complete model, it is necessary to introduce the effect of *T. sinensis* on *D. kuriphilus*: parasitized larvae of the gall wasp will not give rise to adults. Therefore, we rewrite the emergence rate of *D. kuriphilus* as follows:

$$\begin{array}{c} \text{emergence}\;\;\text{rate}=\frac{\eta V_{n-1}\left(T_{d}\right)-Q_{n}\left(T_{t}\right)}{T_{d}} \end{array}$$ (11)

This results in the complete model described by Eq (12)

$$\begin{array}{c} \left\{ \begin{array}{c} {\dot{p}}_{n}\left(\tilde{t}\right)=-\frac{1}{\tau }\left(v_{n-1}\left(1\right)-q_{n}\left(\tilde{t}\right)\right)p_{n}\left(\tilde{t}\right)\\ {\dot{q}}_{n}\left(\tilde{t}\right)=\frac{E_{t}}{\tau }\left(v_{n}\left(1\right)-q_{n}\left(\tilde{t}\right)\right)p_{n}\left(\tilde{t}\right)\\ {\dot{u}}_{n}\left(\tilde{t}\right)=-\mu \left(2-v_{n}\left(\tilde{t}\right)\right)u_{n}\left(\tilde{t}\right)+v_{n-1}\left(1\right)-q_{n}\left(\eta \tau \right)\\ {\dot{v}}_{n}\left(\tilde{t}\right)=E_{d}\mu \left(1-v_{n}\left(\tilde{t}\right)\right)u_{n}\left(\tilde{t}\right) \end{array}\right. \end{array}$$ (12)

with the initial conditions

$$\begin{array}{c} \left\{ \begin{array}{c} p_{n}\left(0\right)=q_{n-1}\left(\eta \tau \right)\\ q_{n}\left(0\right)=0\\ u_{n}\left(0\right)=0\\ v_{n}\left(0\right)=0 \end{array}\right. \end{array}$$ (13)

Eq (12) describes the evolution of the densities of the *D. kuriphilus* and *T. sinensis* over time in a region where the two populations are homogeneously distributed, from which there are no insect fluxes incoming or outgoing.

### Numerical simulations

The system of ordinary differential equations, described by Eq (12), was solved numerically by using a Dormand-Prince 4th/5th order, adaptive stepsize method [26, 27]. The developed computational application is executed in a Windows system of an HPC cluster with cores AMD EPYC 7351 CPU and 64 GB of RAM located at Instituto Politécnico de Bragança (IPB).

The model parameters are *E*_*d*_ = *ηN*_*d*_, *E*_*t*_ = *γN*_*t*_, *τ* = *T*_*t*_/(*ηT*_*d*_) and *μ* = *T*_*d*_/*a*. The survival rate during overwintering of *D. kuriphilus* (*η*) and of *T. sinensis* females (*γ*) are the most uncertain parameters. Depending on the value of these parameters, several types of evolution are possible (see [11]). Research work in Portugal, based on analysis of the content of chestnut galls harvested in the Portuguese regions of Minho and Trancoso, points to values of *η* < 0.9 and *γ* > 0.45 [16]. These observed threshold values are accounted for by two observational facts. Firstly, in Portugal there is a very diverse assemblage of native parasitoids associated with *D. kuriphilus*, most of them also associated with other tree gall such as the oak gall. Secondly, *T. sinensis* is not parasitized by these parasitoids and, in some observed cases, it presents an extended diapause that helps circumventing the effects of a harsh winter [16]. On the other hand, as mentioned in the Introduction, due to its sexual reproduction, only at most 50% of the eggs may eventually emerge as adult female, giving an upper bound *γ* < 0.5. Thus the value of *γ* is fairly well constrained (0.45 < *γ* < 0.50). In all the simulations performed, we considered *γ* = 0.47. The value of *η*, instead, is not, therefore we shall explore the dynamics with different possible values of this parameter.

Regarding the number of eggs laid by *D. kuriphilus* and *T. sinensis*, the observations carried in Portuguese orchards by one of us (A. Bento) point to average values of *N*_*d*_ = 150 and *N*_*t*_ = 71, respectively. These values are similar to those obtained in studies carried out in America and Japan, from wasps emerging from galls collected in chestnut orchards [25, 28]. The non-dimensional length of the egg deposition season of *T. sinensis* is simplified to *τ* ≈ 1/*η*, *i.e*, we consider that the durations of the egg deposition season of the two species are equal. The length of the egg deposition season and the adult lifespan for *D. kuriphilus* are set to *T*_*d*_ = 40 and *a* = 4 days, respectively. These are the values reported by official entities for Europe [20], that are in the range of values observed in the field in Portugal [16]. As a result, the ratio of lengths of the season and individual lifespan for *D. kuriphilus* is set to *μ* = 10.

**Fig 4:**
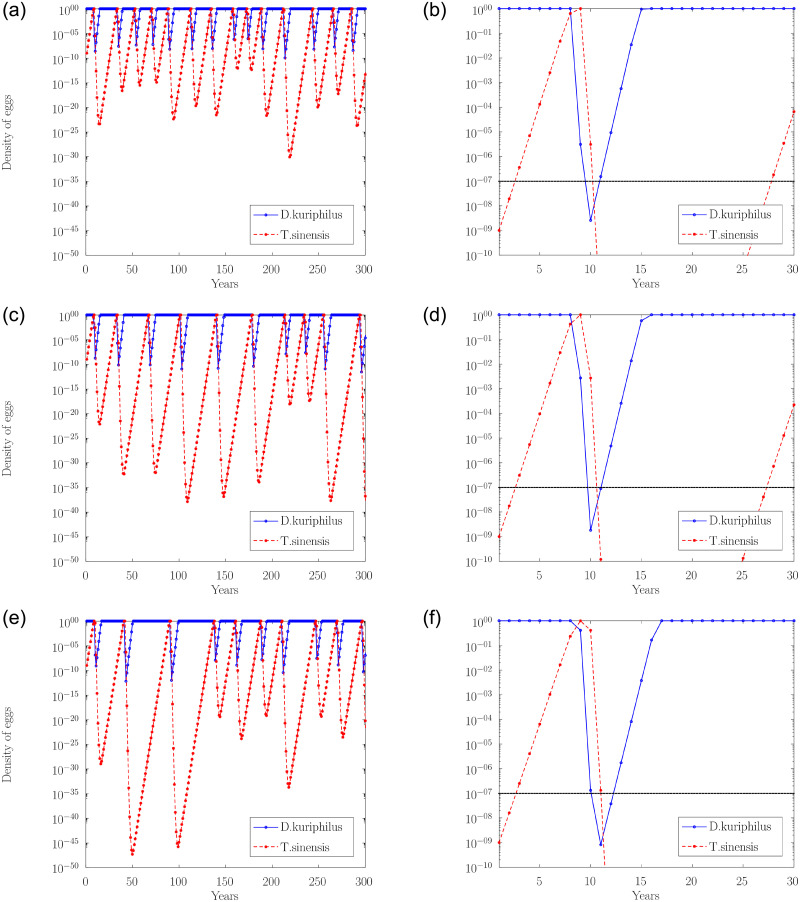
Temporal evolution of the egg density in function of the overwintering survival rate of *D. kuriphilus*. A logarithmic scale is used to highlight the low density reached by the two insect species.

Fig 4 depicts the evolution of the egg density of the two species along the time, in years, for different values of gall wasp overwintering survival rates (*η* = 0.85, *η* = 0.75 and *η* = 0.65). In left (Fig 4a), 4c) and 4e)) over an extended period of 300 years and in the right (Fig 4b), 4d) and 4f)) over the initial period corresponding to the first 30 years after the release of the parasitoid is detailed. The initial conditions correspond to the release of a few individuals of *T. sinensis* per square kilometer in a large chestnut forest infested by *D. kuriphilus* (*v*_*n*_ = 1 and *q*_*n*_ = 10^−9^). Note that actual release protocols dictate much higher densities (e.g. 40 pairs/ha), localized on restricted gall-rich areas, rather than uniformly spread in all of a chestnut-producing area [29].

Additionally, in Fig 4b), 4d) and 4f) it is included the gall wasp survival threshold (horizontal dashed line), that corresponds to having at least one insect per hectare. A rough approximation of this threshold can be obtained considering that the maximum number of shoots per hectare is about 2 × 10^6^ (*β*_*max*_ ≈ 2 × 10^6^ buds ha^−1^). This number assumes that a full-grown chestnut tree in spring produces about 10^4^ buds and that typical production orchards have a density of 100-200 trees per hectare. Although these values are characteristic of Italian chestnut orchards [30], we believe they are also valid for Portugal, as there is no reason for them to be different. Also considering that the average number of eggs laid per bud is 5, as observed by Santos et al. [16] in Portugal, the maximum number of eggs per hectare is around 10^7^ (*V*_max_ ≈ 10^7^ eggs ha^−1^). Therefore, nondimensional densities *v*_*n*_ = 10^−7^ corresponds to less than one insect per hectare. For an isolated, hectare-wide orchard, this would be the extinction threshold. For a chestnut woodland spanning *h* hectares the threshold would be 10^−7^/*h*.

The dynamic of the evolution observed in Fig 4a), 4c) and 4e) is characterized by predator-prey cycles. The two species coexists around an unstable fixed point and therefore the insect egg densities fluctuate from year to year. Cyclical increases and decreases of both species, in which maxima and minima of *T. sinensis* follow the maxima and minima of *D. kuriphilus*. The fluctuations are not periodic, however the cycles are characterized by a fairly well-defined time scale.

Fig 4b), 4d) and 4f) show that during the first seven years the population of *T. sinensis* grows steadily from the very low initial density, while the population of *D. kuriphilus* remains essentially unaffected by the presence of the parasitoid. In the eighth year *q*_*n*_ approaches 1, then *v*_*n*_ begins to decline. Then, in the turn of 1 − 3 years *D. kuriphilus* peaks and, consequently, also the population of *T. sinensis* drops in the following years. The recovery of *D. kuriphilus* occurs in 6 − 7 years, starting from minimum densities that is close to 10^−9^.

On the other hand, Fig 4a), 4c) and 4e) show that the population of *T. sinensis* continues to drop until the recovery of *D. kuriphilus* is almost complete, then it starts to increase. By this time the density of *T. sinensis* may have reached densities almost as low as 10^−30^ (Fig 4e)). From then on, the densities of the two species oscillate in long cycles, with several decades. The decline and the subsequent recovery of *T. sinensis* span almost all the length of the cycle. The high densities of the parasitoid occur only for a few years, while the gall wasp’s maximum density remains for several decades.

Comparing Fig 4a), 4c) and 4e) we observed that the reduction in the gall wasp’s overwintering survival rate has the consequence of increasing the average duration of the cycles, especially in the first 150 years, as well as decreasing the minimum values of population densities, especially of the parasitoid. The population density of *T. sinensis* reaches absurdly low values, which may be close to 10^−50^ in the case of *η* = 0.65.

Fig 4 shows that when the egg density of *T. sinensis* becomes close to 1, then the egg density of *D. kuriphilus*, in a short number of season, drops to values smaller than 10^−7^. The subsequent recovery of *D. kuriphilus* requires several years, during which the population of *T. sinensis*, for lack of deposition sites, decreases to very low values. Cycles with such low minimums do not make sense in nature. In reality, the mathematical model is stating that *T. sinensis*, after the initial transient, wipes out the local population of *D. kuriphilus*, and then becomes extinct itself. The wasp population is considered extinct when its density drops below the survival threshold (horizontal dashed line in Fig 4b), 4d) and 4f)), that corresponds to having at least one insect per hectare.

Comparing Fig 4b), 4d) and 4f), we verify that in the three cases (*η* = 0.85, 0.75 and 0.65) the gall wasp population density is reduced below the survival threshold. This reduction is faster for high *η* values and slightly longer for low *η* values. From the moment the *T. sinensis* density reaches values greater than 0.1, the *D. kuriphilus* density is reduced below the survival threshold in two seasons if *η* = 0.85 or *η* = 0.75, and in three seasons if *η* = 0.65. There is also a small reduction in the minimum value reached by the *D. kuriphilus* population density as *η* decreases (greater than 10^−9^ for *η* = 0.85 or *η* = 0.75, and less than 10^−9^ for *η* = 0.65).

The spatially-homogeneous model predicts the local extinction of the pest. However, this mathematical model is deterministic and as such does not take into account the random events that occur especially when population densities are low. Under these circumstances, the gall wasp is more likely to survive than the parasitoid, because the probabilities finding chestnut buds is much higher than the probability of the parasitoid finding the wasp galls. More generally, we must stress that our model does not take into account the presence of refugia, which, by the nature of the trophic relationships under study, appear to be more likely to exist for *D. Kuriphilus*. In fact, when the density of gall wasp eggs has dropped to very low values (but still above the extinction level) finding a place where to lay eggs becomes exceedingly difficult for *T. sinensis*. If the density of *T. sinensis* happens not to be very high, then there’s a chance that a handful of galls might be missed by random factors as, for instance, a branch cracks and covers a gall, thereby effectively occulting it to the *T. sinensis*, or something like that. These would be refugia, where a tiny number of gall wasps might survive. The next year, *T. sinensis* would be locally extinct, but there would still be a handful of gall wasps around, ready to re-colonize the orchard. In other words, if the dynamics is such that at year *n* are possible very low densities of both *T. sinensis* and the gall wasp, then there’s a chance (just by random events) that at year *n*+1 *T. sinensis* will be locally extinct, but the gall wasp will be not.

On the other hand, the model assumes populations homogeneously distributed in space, which is unlikely to occur in the field, except in small orchards, due mainly to the different dispersion capacities of the two species.

## Space-dependent model

Adding a spatial dependence to the previous homogeneous model will take into account the fact that the two species move over time from one area to another. To accomplish that we add diffusion terms to the adult populations in the model described by Eq (12).

### Mathematical model

We assume that the random motion of individual insects within a wood at spatial scales large enough that the population density may be approximated by a continuous, smooth field, will produce a Fourier–Fick flux, that is, a flux proportional to the gradient of the insect population density. This gives rise in the equations for the *P*_*n*_ and *U*_*n*_ to terms proportional to the Laplacian of these two fields [31]. The proportionality constants, *D*_*d*_ and *D*_*t*_, respectively for the flux of *D. Kuriphilus* and *T. sinensis* are called diffusion coefficients. By taking as unit of length the quantity ${\left(D_{d}T_{d}\right)}^{\frac{1}{2}}$, and defining the diffusivity ratio *δ* = *D*_*t*_/*D*_*d*_, we may bring the new terms to non–dimensional form, resulting in the following spatially–dependent system of equations:

$$\begin{array}{c} \left\{ \begin{array}{c} \frac{\partial }{\partial \tilde{t}}p_{n}\left(x,y,\tilde{t}\right)=\delta \nabla^{2}p_{n}\left(x,y,\tilde{t}\right)-\frac{1}{\tau }\left(v_{n-1}\left(x,y,1\right)-q_{n}\left(x,y,\tilde{t}\right)\right)p_{n}\left(x,y,\tilde{t}\right)\\ \frac{\partial }{\partial \tilde{t}}q_{n}\left(x,y,\tilde{t}\right)=\frac{E_{t}}{\tau }\left(v_{n}\left(x,y,1\right)-q_{n}\left(x,y,\tilde{t}\right)\right)p_{n}\left(x,y,\tilde{t}\right)\\ \frac{\partial }{\partial \tilde{t}}u_{n}\left(x,y,\tilde{t}\right)=\nabla^{2}u_{n}\left(x,y,\tilde{t}\right)-\mu \left(2-v_{n}\left(x,y,\tilde{t}\right)\right)u_{n}\left(x,y,\tilde{t}\right)+v_{n-1}\left(x,y,1\right)-q_{n}\left(x,y,\eta \tau \right)\\ \frac{\partial }{\partial \tilde{t}}v_{n}\left(x,y,\tilde{t}\right)=E_{d}\mu \left(1-v_{n}\left(x,y,\tilde{t}\right)\right)u_{n}\left(x,y,\tilde{t}\right) \end{array}\right. \end{array}$$ (14)

with the initial conditions

$$\begin{array}{c} \left\{ \begin{array}{c} p_{n}\left(x,y,0\right)=q_{n-1}\left(x,y,\eta \tau \right)\\ q_{n}\left(x,y,0\right)=0\\ u_{n}\left(x,y,0\right)=0\\ v_{n}\left(x,y,0\right)=0 \end{array}\right. \end{array}$$ (15)

### Numerical simulations

In each season *n*, the system (14), with the initial conditions given by Eq (15), is solved in two steps. Firstly, the two equation for *T. sinensis* are solved in order to determine the distribution of the eggs deposited (*q*_*n*_). This quantity will influence the amount of adult gall wasps that will hatch, so it goes into the *u*_*n*_ equation. Then the gall wasp equations are solved, allowing to determine the distribution of laid eggs (*v*_*n*_) during the season *n*. In the next season, this quantity will enter the equation of *p*_*n*_, through the term *v*_*n*−1_. The computational algorithm repeats this procedure each year and, consequentially, enables to look at the spatial evolution of the density of *D. kuriphilus* and *T. sinensis* over the season and over years.

The two sets of partial differential equations, described by Eq (14), are solved by means of the Matlab PDE Toolbox, where these equation are discretized by a finite element method and the resulting system of ordinary differential equations is solved with the built-in Matlab function ode15 (for details, see [32]). The finite element mesh of the 2-D domain created with Matlab uses triangular finite elements, defined by three nodes, with linear interpolation functions. The upper bound on the mesh edge lengths is set to 0.1.

The algorithm implemented in built-in Matlab function ode15 is based on the discretization of the time derivative by numerical differentiation formulas (NDFs) of orders 1 to 5. The time step is defined internally in order to ensure convergence. In each time step, the non-linear system of algebraic equation is solved through Newton-Raphson method (for more details see [33]). The developed computational application is executed in a Windows system of an HPC cluster with cores AMD EPYC 7351 CPU and 64 GB of RAM located at Instituto Politécnico de Brangança (IPB).

The diffusivity ratio between the two species (*δ* = *D*_*t*_/*D*_*d*_) is, jointly with the overwintering survival rates (*η* and *γ*), the most important parameters of this mathematical model. It was observed that *D. kuriphilus* moves faster than *T. sinensis* and travels longer distances [34]. In Portugal, it was reported that *D. kuriphilus* can disperse through active fly, with an average rate of dispersion estimated between 8-25 km by season [16]. Considering *η* = 0.65 and an annual displacement of 8 km, according to the approximate expression of the *D. kuriphilus* front speed, proposed by Paparella et al. [11], the diffusion coefficient value is *D*_*d*_ = 0.889 km^2/^d.

Regarding *T. sinensis*, it is common to assume that its dispersion capacity is much lower than that of the *D. kuriphilus* and does not exceed 2 km per year, as reported by Toda et al. [19] for Japan. The diffusion coefficient corresponding to this propagation speed is *D*_*t*_ ≈ 0.016 km^2/^d giving a diffusivity ratio of *δ* ≈ 0.018.

**Fig 5:**
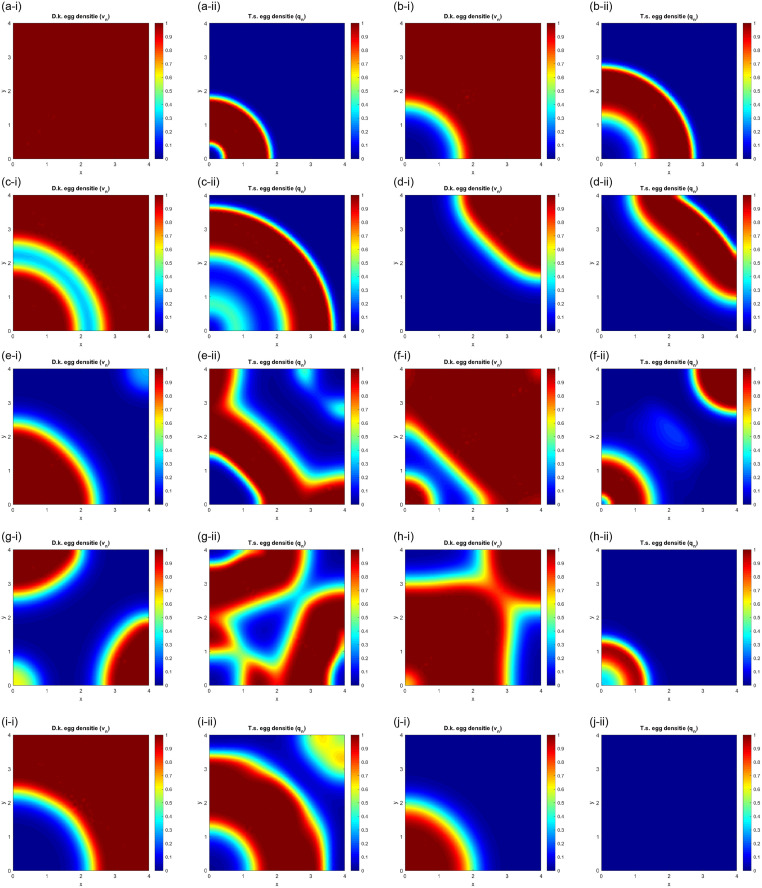
Spatial evolution of the *D. kuriphilus* and *T. sinensis* egg density over the years, in an area of 4 × 4 non-dimensional length units.

The results of the numerical solution of the space-dependent model given by Eq (14) are presented in the time sequence of Fig 5 for the years 3, 6, 9, 12, 18, 21, 24, 27, 30 and 33 after the *T. sinensis* release. As initial conditions, it is considered that *T. sinensis* is released close to the lower-left corner of a square forest (the size of 4 × 4 non-dimensional units corresponds in this numerical solution to a physical size of approximately 22 × 22 km^2^), completely infested by *D. kuriphilus*, *i.e*, the initial density of *D. kuriphilus* is set to one everywhere, and the initial density of *T. sinensis* is zero everywhere except at the lower-left corner where it is maximal. In the numerical model, null Neumann boundary conditions are imposed; the area is considered insulated, *i.e*, there is no flux of insects in or out of the computational domain. It is assumed *δ* = 0.018, *η* = 0.65 and *γ* = 0.47 and the remaining parameters retain the same values previously used in the homogeneous model, described by the Eq (12). For each year, the Figs on the left represents the density of *D. kuriphilus* eggs, while the Figs on the right represents the density of *T. sinensis* eggs.

As shown in Fig 5, *T. sinensis* initially spreads radially like a wave, leaving a roughly circular area of the forest free of both the pest and the parasitoid, which is recolonized by the pest a few years after the release. The parasitoid front moves in the same direction until it sweeps the entire area. After that it moves in the opposite direction and attacks the area that has since been recolonized by the pest. The parasitoid population then splits in two parts, one located at the end of the forest and the other at the release site (year 21). These fronts hunts the pest separately in the two other corners of the area and then they merge into a single front located at the starting point which begins to spread radially, occupying a large part of the area (year 30). At that time the pest is reduced to very low density in almost the entire area and, as a result, in the following years the parasitoid disappears completely from the forest (year 33).

Fig 5 shows a cyclic phenomenon that moves in the area over time in consequence of the continuous alternation of local extinctions and recolonizations. The reappearance of the pest in areas previously swept by the parasitoid occurs because *D. kuriphilus* has higher diffusivity than *T. sinensis*. The latter cannot avoid recolonization behind its front of maximum concentration. In this area, previously swept by *T. sinensis*, its concentration is too low to prevent *D. kuriphilus* from reappearing. On the other hand, due to its low diffusivity, the parasitoid is unable to move from its maximum concentration area in time to avoid recolonization by the pest.

**Fig 6:**
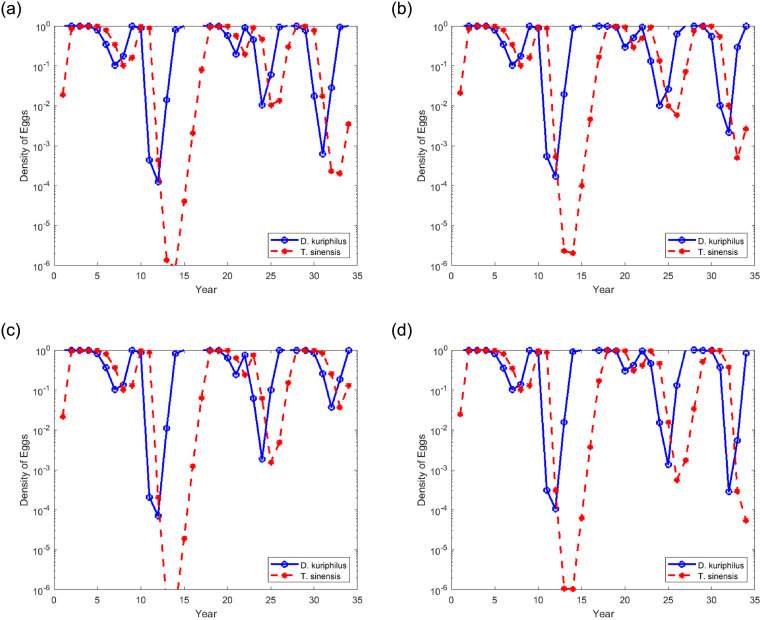
Egg density of *D. kuriphilus* (*v*_*n*_) and *T. sinensis* (*q*_*n*_) in a given place of the forest over the time.

The local evolution of the two species, considering their dispersion capacities, makes it possible to make a comparison with the results obtained with the homogeneous model, illustrated in Fig 4 in which the dispersion of the two species was not taken into account. In Fig 6, the density of the eggs of two species at the end of each season, in the position *x* = 1 and *y* = 1, located close to the lower left corner of the area represented in Fig 5, is traced over time for two different values of the total area (4 × 4 and 5 × 5) and two different values of overwintering survival rate of *D. kuriphilus* (*η* = 0.65 and *η* = 0.85).

Fig 6 shows that the cycles already observed in the case of homogeneous solution, shown in Fig 4, have disappeared. In general, the variations in densities have much smaller amplitudes. Variation of the *D. kuriphilus* density is, in general, closely followed by the variation of the parasitoid density. After starting to decrease, the *D. kuriphilus* density manages to recover despite the relatively high numbers of the parasitoid. This is due to the high dispersion capacity of the gall wasp, which allows recolonizing areas previously swept by the parasitoid. As a result, its density does not decrease much at that location and quickly returns to 1, causing, in turn, an increase in the density of the parasitoid. The recovery time depends on the minimum value reached by *D. kuriphilus*. The lower this value is, the more years *T. sinensis* takes to recover. The lower value reached by *D. kuriphilus* is close to 10^−4^ which is well above the extinction threshold.

Comparing the results obtained with the non-dimensional areas 4 × 4 (s Fig 6a) and 6c)) and 5 × 5 (Fig 6b) and 6d)), it can be seen that the increase in the forest area has the effect of slightly increasing the minimum values reached by the densities of the two species. On the contrary, the increase in the overwintering survival rate of *D. kuriphilus* from 0.65 to 0.85 results in more pronounced drops in the density values of the two species.

**Fig 7:**
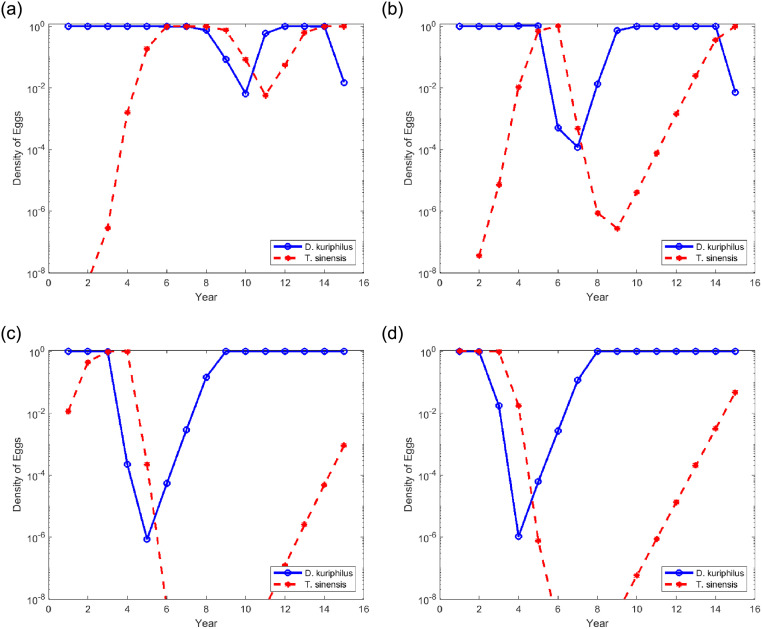
Egg density of *D. kuriphilus* (*v*_*n*_) and *T. sinensis* (*q*_*n*_) at a fixed position in the computational domain as a function of time, after multiple simultaneous releases of *T. sinensis* performed at year 0 from stations placed on a regular mesh.

Fig 7 shows the density of the eggs of two species at the end of each season, at the position *x* = 1 and *y* = 1 of the area represented in Fig 5, after multiple simultaneous releases of *T. sinensis* are performed at year zero from stations placed on a regular mesh with different sizes. For Fig 7a) the release stations are placed at the 4 corners of the square simulated forest area. The mesh is then progressively refined by arranging the release stations at the vertices of a 2 × 2 square pattern in Fig 7b), 3 × 3 in Fig 7c), and 4 × 4 in Fig 7d). The distance between neighboring release stations is thus of 4, 2, 4/3, and 1 non–dimensional units. One non-dimensional unit corresponds approximately to 5.5 km. In all these simulations the overwintering survival rate is set to *η* = 0.65.

The observation of Fig 7 indicates that in general the minimum value reached by the density of gall wasp eggs decreases with the increase in the number of releases. It is also verified that this minimum value does not fall below 10^−6^ despite the mesh refinement. This result shows that there is no point in increasing the number of *T. sinensis* releases, the local extinction of *D. kuriphilus* is not achieved.

**Fig 8:**
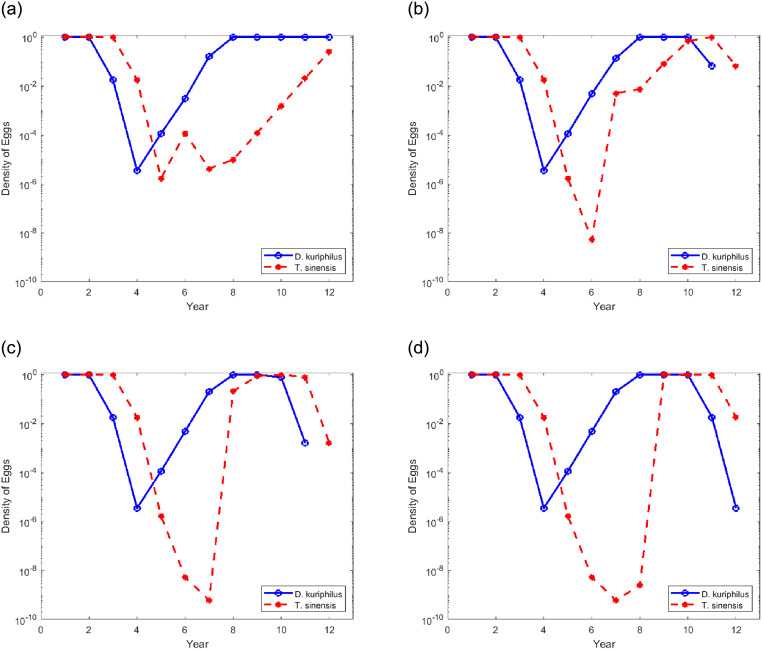
Egg density of *D. kuriphilus* (*v*_*n*_) and *T. sinensis* (*q*_*n*_) in a given place of the forest over the time after several releases of *T. sinensis* made simultaneously, at years 1 and *n*_*r*_ according to a regular mesh 4 × 4.

Fig 8 shows the evolution of the density of the eggs of two species at the end of each season, in the position *x* = 1 and *y* = 1 of the area represented in Fig 5, that corresponds to a point in the 4 × 4 grid where *T. sinensis* was released in years 1 and is newly released in year *n*_*r*_ = 6,7,8,9. In all these simulations the overwintering survival rate is set to *η* = 0.65.

The results observed in the Fig 8 show that there are no benefits, in terms of biological pest control, in making new releases of the parastoid when the gall wasp egg density is low. Fig 8a) shows that the injection made in year 6, after the first release, only contributes so that the density of *T. sinensis* does not fall to a very low level. This new release only slightly accelerates the reconstitution of the parasitoid population and, as such, it does not prevent the *D. kuriphilus* population from implanting in the place for a long period. Fig 8a)–8c) shows that when *T. sinensis* is released in years when the gall wasp eggs density is above 10^−2^ (level of infestation is 1%), the maximum *D. kuriphilus* implantation period is reduced to three years.

**Fig 9:**
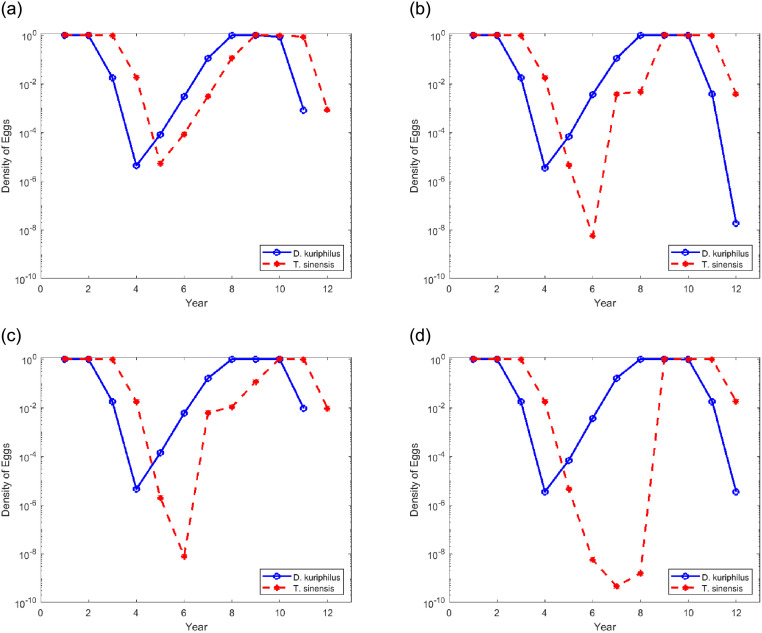
Egg density of *D. kuriphilus* (*v*_*n*_) and *T. sinensis* (*q*_*n*_) in a given place of the forest over the time after several release of *T. sinensis* made simultaneously with periodicity *k*_*r*_ according to a regular mesh 4 × 4.

It is possible to think that the periodic release of *T. sinensis* can contribute to the eradication of the pest or at least to keep the level of infestation low. The results shown in the Fig 9 do not support this assumption. Fig 9 shows the evolution of the density of the eggs of both species at the end of each season, in the position *x* = 1 and *y* = 1 of the area represented in Fig 5, that corresponds to a point in the 4 × 4 grid where *T. sinensis* is released every *k*_*r*_ years, considering *η* = 0.65.

In all the cases illustrated in Fig 9, the gall wasp is able to implant itself again and infest the forest for a period of three years, regardless of whether the parasitoid is released every year or every 4 years.

Comparing Fig 9 with Fig 8, it appears that it is preferable to release *T. sinensis* in specific years instead of periodically. For the biological control action to be effective, the releases must be made when the level of infestation of trees by *D. kuriphilus* is close to 1%. For this purpose, it is necessary to collect over the years, field information that allows monitoring the evolution of the population density of the gall wasp.

## Final considerations

A spatially-homogeneous model shows that biological control, under optimal conditions, can be successful. This result suggests that for small, isolated chestnut orchards, biological control should be effective. For this, it is necessary that the release of *T. sinensis* is performed when the area is close to be fully infested, *i.e.*, when the *D. kuriphilus* population density is close to its maximum value. In these conditions and considering spatially homogeneous populations of *D. kuriphilus* parasitized by *T. sinensis*, the parasitoid is able to implant itself in the domain and, within a few years, to eradicate the pest, extinguishing itself in turn afterwards. These results show that biological control of *D. kuriphilus* can be a very promising option in the case of chestnut trees plantations that occur in small extensions well separated from each other.

The presence of an important number of native parasitoids that parasitize *D. kyriphilus* in Portugal does not affect significantly biological control using *T. sinensis*. The simulations executed with different values of *D. kuriphilus* overwintering survival rate show that decreasing this rate causes only a small decrease in the initial minimum value of *D. kyriphilus* population densities. This aspect can benefit biological control.

On the other hand, the results obtained with the spatially–dependent version of the model shows that in large, or heterogeneous, areas biological control is likely to fail in achieving the desired goal of eradicating the pest. This occurs because *D. kuriphilus* has a higher dispersion capacity than *T. sinensis*.

This allows *D. kuriphilus* to recolonize areas previously swept by *T. sinensis* where both species have become locally extinct. In these circumstances, some level of biological control would be achieved by many simultaneous releases of *T. sinensis* along a regular grid with a spacing of about 7 km (Fig 7c)). Closer release points do not seem to improve pest control effectiveness.

This procedure does not succeed in fully eradicating *D. kuriphilus*, although it does reduce the level of infestation to very low rates over a few years. It is, therefore, necessary to perform new releases of the parasitoid in order to minimize the impact caused by the reconstruction of the gall wasp population. Very frequent releases of *T. sinensis* are not efficient. It is preferable to perform releases in years when the level of infestation approaches (or exceeds) 1%.

In Portugal, vast chestnut–trees woods are not very frequent. However, there are regions with extensive chestnut production (Bragança, Vila Real, Viseu, Guarda and Portalegre), in many small orchards next to each other. In these regions it is preferable to follow a collective strategy for the entire region, otherwise the gall wasp will move between chestnut orchards and reconstruct its population uninterruptedly.
