# Correction: Biological control of the Asian chestnut gall wasp in Portugal: Insights from a mathematical model

**DOI:** 10.1371/journal.pone.0257990

**Published:** 2021-09-23

**Authors:** Carlos Balsa, Albino Bento, Francesco Paparella

Figs 4, 5, 6, 7, 8 and 9 are incorrect. The publisher apologizes for the errors. The authors have provided corrected versions here.

**Fig 4:**
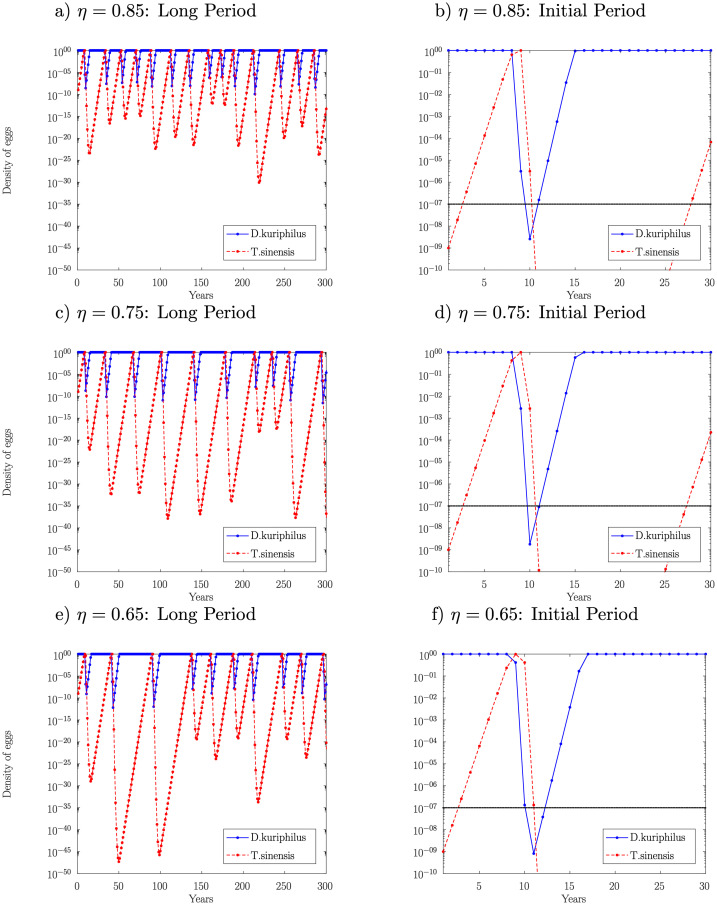
Temporal evolution of the egg density in function of the overwintering survival rate of *D*. *kuriphilus*. A logarithmic scale is used to highlight the low density reached by the two insect species.

**Fig 5:**
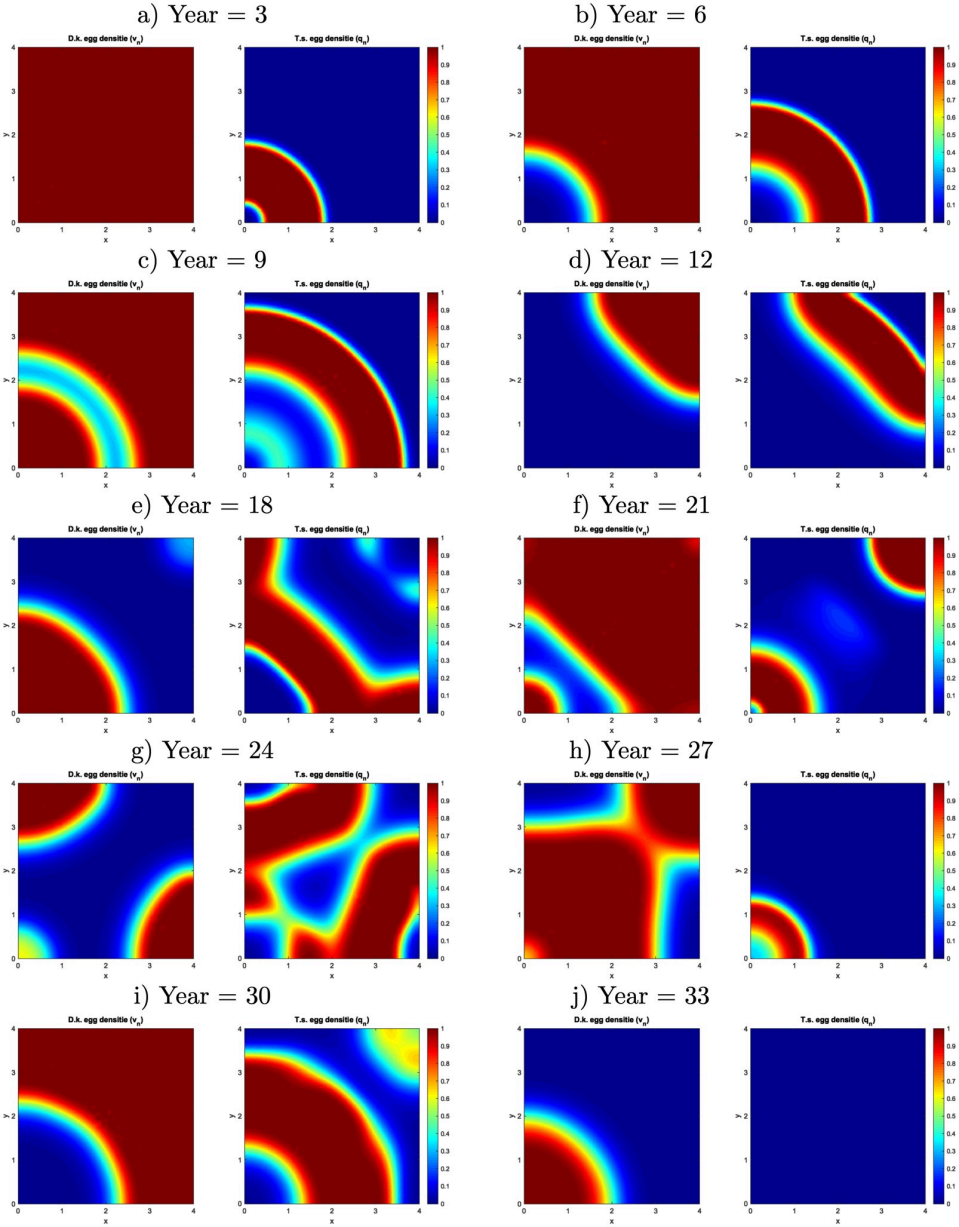
Spatial evolution of the *D*. *kuriphilus* and *T*. *sinensis* egg density over the years, in an area of 4 × 4 non-dimensional length units.

**Fig 6:**
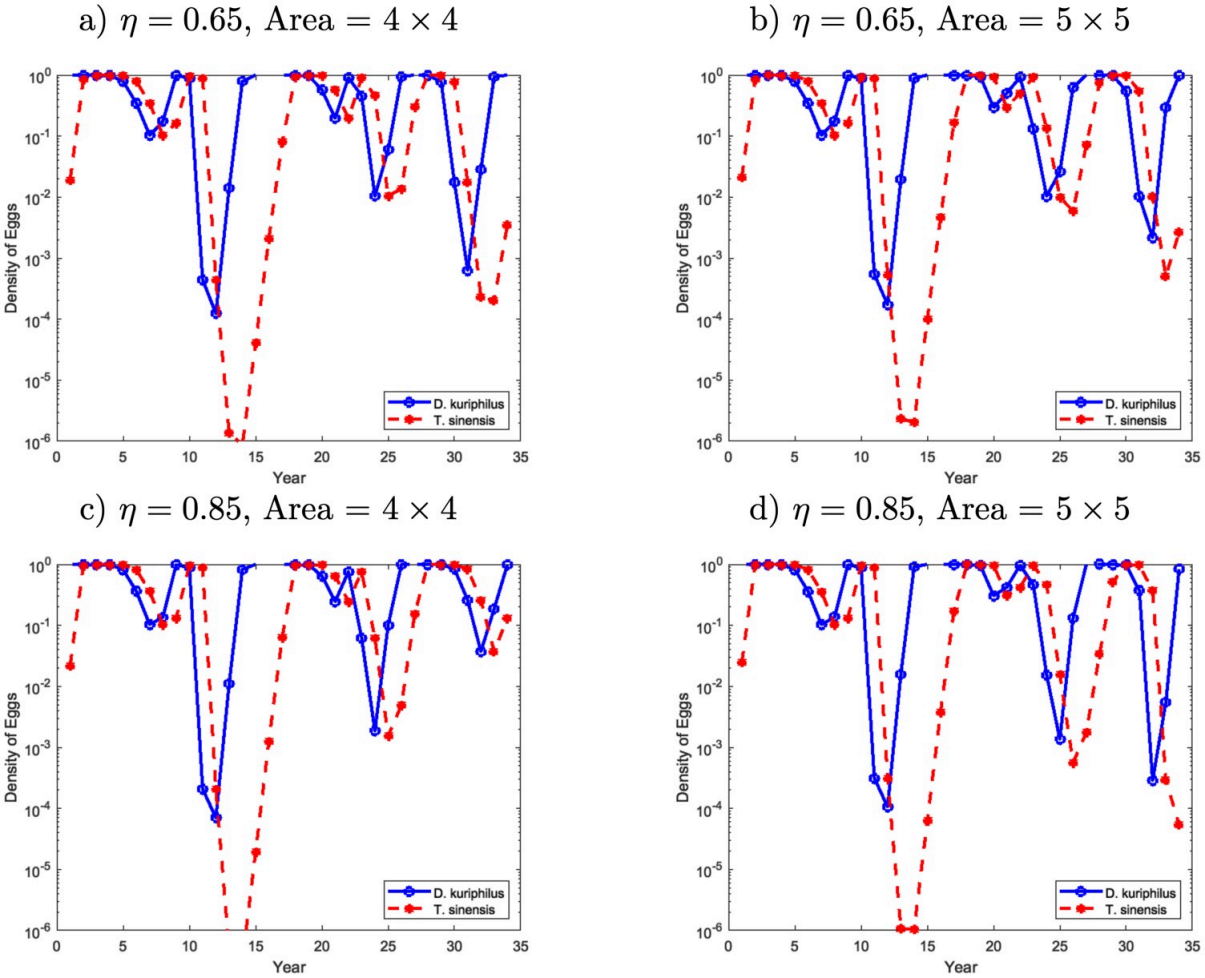
Egg density of *D*. *kuriphilus* (*v*_*n*_) and *T*. *sinensis* (*q*_*n*_) in a given place of the forest over the time.

**Fig 7:**
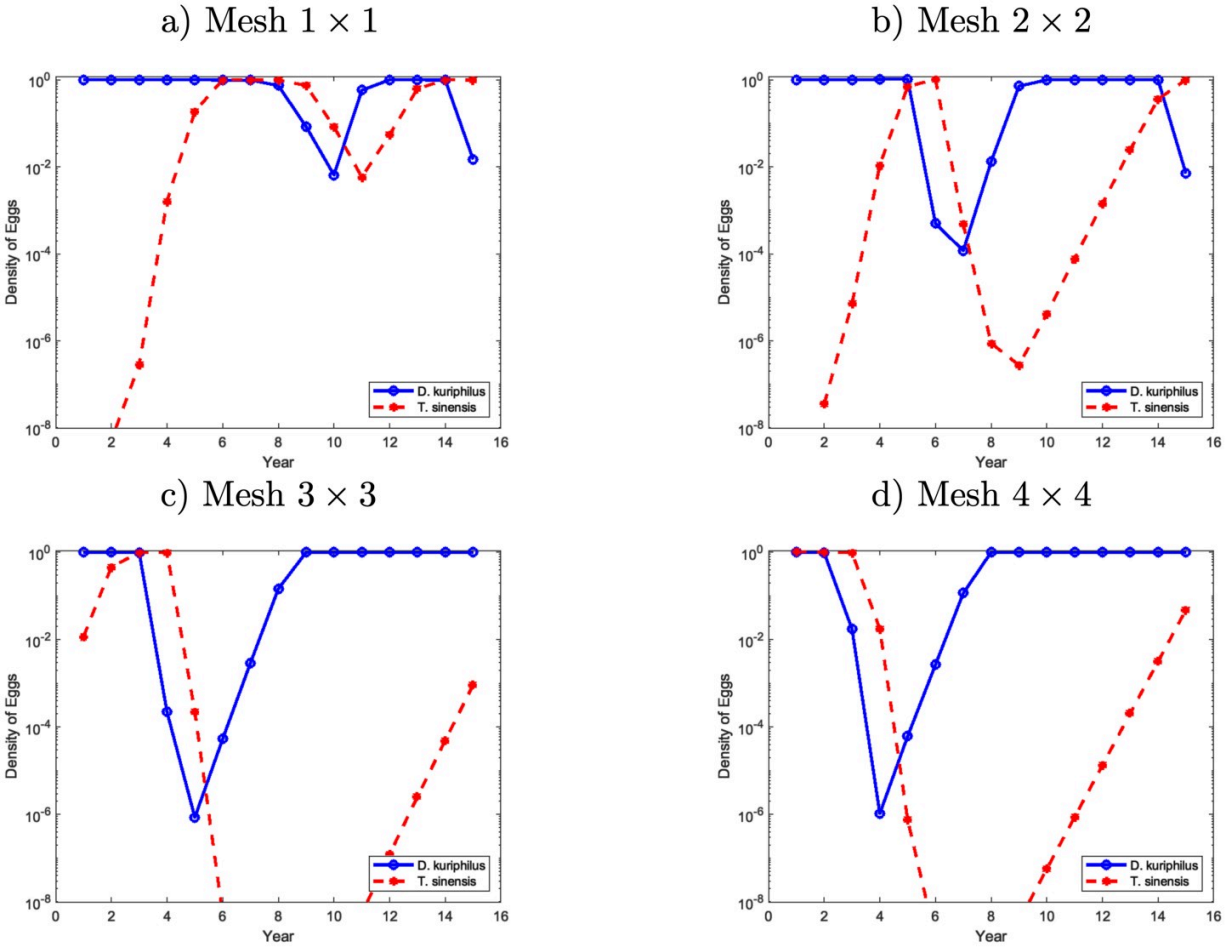
Egg density of *D*. *kuriphilus* (*v*_*n*_) and *T*. *sinensis* (*q*_*n*_) at a fixed position in the computational domain as a function of time, after multiple simultaneous releases of *T*. *sinensis* performed at year 0 from stations placed on a regular mesh.

**Fig 8:**
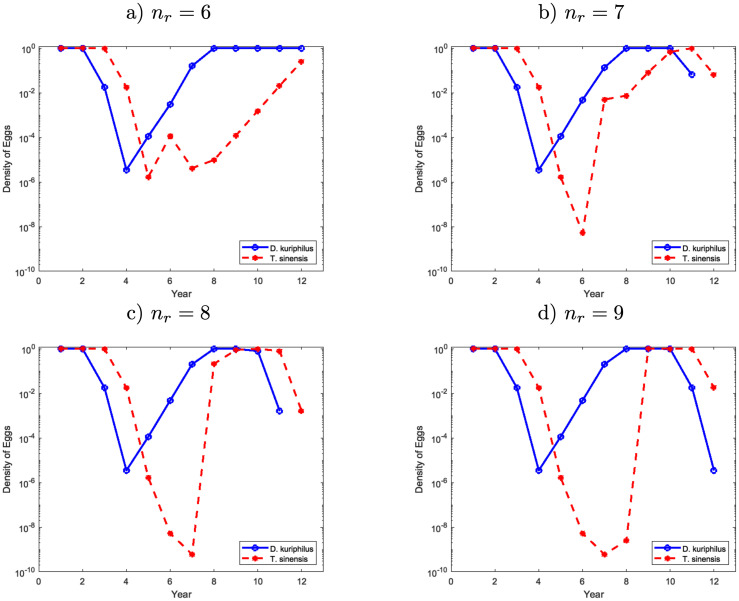
Egg density of *D*. *kuriphilus* (*v*_*n*_) and *T*. *sinensis* (*q*_*n*_) in a given place of the forest over the time after several releases of *T*. *sinensis* made simultaneously, at years 1 and *n*_*r*_ according to a regular mesh 4 × 4.

**Fig 9:**
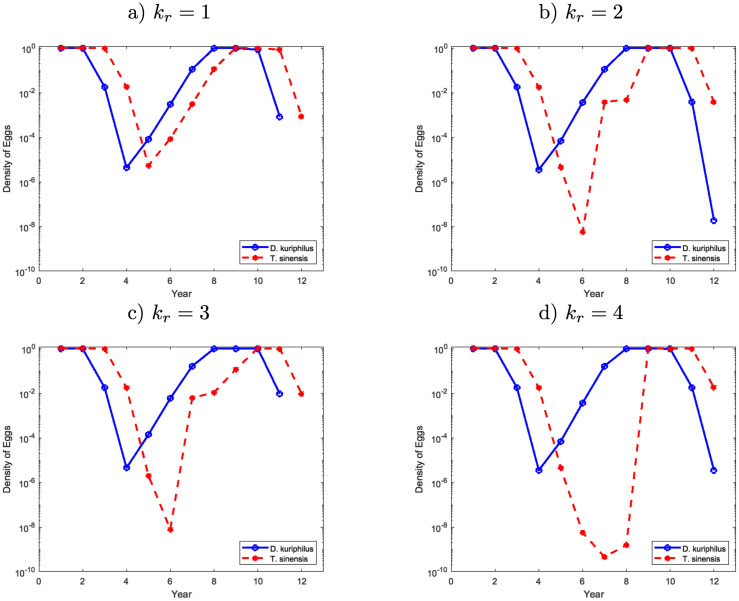
Egg density of *D*. *kuriphilus* (*v*_*n*_) and *T*. *sinensis* (*q*_*n*_) in a given place of the forest over the time after several release of *T*. *sinensis* made simultaneously with periodicity *k*_*r*_ according to a regular mesh 4 × 4.
